# Vasculitis, Autoimmunity, and Cytokines: How the Immune System Can Harm the Brain

**DOI:** 10.3390/ijerph18115585

**Published:** 2021-05-24

**Authors:** Alessandra Tesser, Alessia Pin, Elisabetta Mencaroni, Virginia Gulino, Alberto Tommasini

**Affiliations:** 1 Department of Pediatrics, Institute for Maternal and Child Health-IRCCS “Burlo Garofolo”, 34137 Trieste, Italy; alessandra.tesser@burlo.trieste.it (A.T.); alberto.tommasini@burlo.trieste.it (A.T.); 2 Department of Pediatrics, Ospedale Santa Maria Misericordia, 06123 Perugia, Italy; elisabetta.mencaroni@ospedale.perugia.it; 3 Family Pediatrician, Valnerina District, UslUmbria2, 06046 Norcia, Italy; virginia.gulino@pls.ulsumbria2.it; 4 Department of Medicine, Surgery and Health Sciences, University of Trieste, 34149 Trieste, Italy

**Keywords:** immune-mediated brain disorders, autoimmunity, autoinflammation, vasculitis, interferons, TNFα, IL-1

## Abstract

More and more findings suggest that neurological disorders could have an immunopathological cause. Thus, immune-targeted therapies are increasingly proposed in neurology (even if often controversial), as anakinra, inhibiting IL-1 for febrile inflammatory illnesses, and JAK inhibitors for anti-interferons treatment. Precision medicine in neurology could be fostered by a better understanding of the disease machinery, to develop a rational use of immuno-modulators in clinical trials. In this review, we focus on monogenic disorders with neurological hyper-inflammation/autoimmunity as simplified “models” to correlate immune pathology and targeted treatments. The study of monogenic models yields great advantages for the elucidation of the pathogenic mechanisms that can be reproduced in cellular/animal models, overcoming the limitations of biological samples to study. Moreover, monogenic disorders provide a unique tool to study the mechanisms of neuroinflammatory and autoimmune brain damage, in all their manifestations. The insight of clinical, pathological, and therapeutic aspects of the considered monogenic models can impact knowledge about brain inflammation and can provide useful hints to better understand and cure some neurologic multifactorial disorders.

## 1. Introduction

Mounting evidence shows that several neurological disorders have an immunopathological cause and, accordingly, immune-targeted therapies are increasingly proposed in neurology. For example, intravenous immunoglobulins or anti-CD20 antibodies have been used in disorders associated with autoantibodies reacting toward brain structures. More recently, blood-brain-barrier (BBB) permeant small molecules have been introduced to clear intracerebral pathogenic B lymphocytes [1]. T cells can be targeted as well by several medications, as in the treatment of multiple sclerosis (MS), even if it is still not clear what is the main player in MS-related brain damage. Among cytokines, a significant focus has been placed on interleukin (IL)-1 in febrile inflammatory illnesses, inhibited by anakinra, and interferons (IFNs), inhibited by JAK inhibitors.

In spite of this evidence, immune-targeted treatments in neurology are often controversial, apart from a few cases in which immune-modulators have been approved by regulatory agencies (Table 1). A better knowledge of the mechanisms involved in each disease is needed to develop rational use of immuno-modulators in controlled clinical trials, fostering precision medicine in neurology. From a pathogenic point of view, immune-mediated neurological diseases can be due to at least four major conditions: monogenic disorders with hyper-inflammation/autoimmunity; post-infection neurological disorders; paraneoplastic autoimmune disorders; idiopathic immune-mediated disorders.

In this review, monogenic disorders with neurological hyper-inflammation/autoimmunity will be discussed as simplified models to correlate immune pathology and targeted treatments (Table 2).

The greatest advantage of models based on monogenic disorders relies on the knowledge of a definite molecular defect, which allows for establishing a clear hierarchy in the pathogenic mechanisms. Moreover, the biological effect of monogenic defects can be studied in cellular and animal models overcoming the limitations of obtaining biological samples to study.

Indeed, the study of multifactorial inflammatory/autoimmune brain disorders in humans is hindered by several shortcomings: it is hard to know the histological and pathological process in the brain, especially in the first phases of the disease; anomalies found in blood or in cerebrospinal fluid (CSF) may reflect differences related to the BBB integrity (which can be impaired by the interaction between endothelial cells and brain macrophages [2,3]) and to whether the primary pathological process arose in the brain or in other organs; it is uncertain how animal models can be relevant to the corresponding human disease; data from autopsies can only highlight late pathological events, being poorly relevant to the initial events in the pathogenic sequence.

Conversely, monogenic disorders provide unique opportunities to study the mechanisms of neuroinflammatory and autoimmune brain damage, from early to late pathogenic phenomena. Recent experience in the field of rheumatology showed that knowledge on multifactorial disorders may highly benefit from studies on their monogenic mimics, as in the case of cryopyrinopathies and Still’s disease or monogenic and multifactorial systemic lupus erythematosus (SLE) or Behçet’s disease (BD) [4,5,6,7,8]. Interestingly, the availability of molecularly targeted medications is providing an indirect tool to establish a possible pathogenic relationship between monogenic and multifactorial disorders. The crucial role of a given molecule in the pathogenesis of a disease can be revealed by proving the effect of specifically targeted inhibitors in correcting or attenuating the typical disease phenotype. For example, Table 1 shows a list of monogenic and multifactorial disorders characterized by the crucial pathogenic involvement of single cytokines. For some disorders, the association with a single cytokine is particularly strong, whilst other disorders have a more complex pathogenesis, even if a hierarchically greater importance can be often attributed to single cytokines. Table 2 lists three groups of monogenic disorders associated with immune-mediated brain damage. We will discuss in detail how clinical, pathological, and therapeutic aspects of these monogenic disorders can impact knowledge about brain inflammation and can provide useful hints to better understand and treat some neurologic multifactorial disorders.

## 2. From Monogenic to Multifactorial: Interferon-Related Brain Disorders

### 2.1. The Role of Type I Interferon: The Immune Response to Nucleic Acids

Cellular sensing of pathogens, such as bacteria, fungi, and viral genomes, is based on the recognition of highly conserved structures, the so-called pathogen-associated molecular patterns (PAMPs), which are easily distinguishable from the host components. Receptors named “pattern-recognition receptors” (PRRs) are constitutively expressed by all cells and have evolved, indeed, to sense PAMPs [16,17] and to put in place the first-line defense, by activation of the innate inflammatory response. Some PRRs are localized on the cell membrane, others inside the cell. PRRs are also involved in sensing damage-associated molecular patterns (DAMPs), “endogenous” molecules that are released upon cellular stress/damage, mainly nucleic acids which act as “danger signals” in promoting strong (and, sometimes, pathogenic) inflammatory response aimed at removing damaged cells, thus preventing potential cell oncogenic transformation, while favoring tissue repair [18].

Triggering an intracellular sensor with “foreign” or “self” nucleic acids results in a tightly controlled inflammatory response dominated by type I IFNs production (IFNα and IFNβ), considered as a powerful, conserved, and sophisticated physiological defense mechanism against viruses and intracellular bacteria [19,20,21,22].

Although the discovery of IFNs dates back to the 1960s [23], several components involved in this signaling pathway have been only described in the last decade: the major intracellular DNA sensor has been identified in the cyclic GMP-AMP synthase (cGAS) [24]. Once activated by cytoplasmic double-stranded nucleic acids, cGAS catalyzes the production of the dinucleotide cyclic GMP-AMP (cGAMP) [25], a second messenger that activates the Stimulator of Interferon Genes (STING). From STING, the signal conveys on the TBK1 kinase (TANK-Binding Kinase 1), that induces the IFNs production acting on the IFN Regulatory Factors IRF3/7 [26,27].

**Figure 1:**
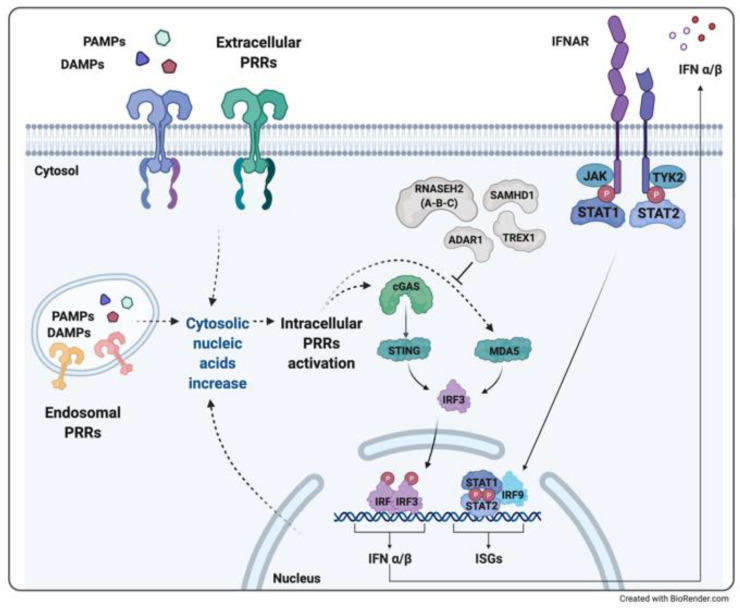
Type I Interferonopathies with neuroinflammation may arise from the gain of function mutations in genes encoding intracellular sensors and adaptors (as *IFIH1* gene coding for MDA5 in Aicardi-Goutières syndrome (AGS), and *STING* in STING-associated vasculopathy with onset in infancy (SAVI)) or loss of function mutations in enzymes involved in nucleotide metabolism which causes an increase of cytosolic DNA (as in AGS due to *TREX1* or *SAMHD1* mutations), RNA/DNA hybrids (as in AGS due to one of the endonucleases of the RNAseH2 complex) or RNA (as in DADA disease, due to *ADAR1* gene). Both mechanisms result in an excessive IRF3-mediated transcription of type I interferons (IFNα/β), which trigger an autocrine and/or paracrine loop of the hyperinflammatory state through IFN-α/β receptor (IFNAR) activation and IFN stimulated genes (ISGs) transcription.

The binding of IFN with its receptor (IFNAR) on the target cells induces a subsequent cascade of phosphorylation and activation of the associated JAK kinases, with the activation and recruitment of a multimeric complex (STAT adaptor molecules and IRF9) that moves into the nucleus, binds specific DNA sequences and determines the transcription of the so-called IFN stimulated genes (ISGs) [28,29,30] (Figure 1).

This defense strategy, however, can represent a double-edged weapon: cytoplasmic accumulation of nucleic acids, whether derived from physically or chemically-induced cellular injury or from pathogen infections, can result in an aberrant inflammatory response. This response can be amplified in the presence of defective nucleic acid disposal machinery, for example, due to deficiency in cellular nucleases. A sustained IFN-mediated inflammation eventually increases dendritic cell activation, plasma cell maturation, and autoantibody production, favoring the development of IFN-associated autoimmunity disorders, as in SLE, dermatomyositis, or Sjogren’s syndrome [31,32,33,34,35,36]. Type I interferonopathies have been recently described as a novel group of Mendelian disorders due to alterations in the recognition and/or disposal of nucleic acids, causing an aberrant stimulation of type I IFNs pathway and disease onset. Sharing significant aspects of the pathogenic cascade, the clinical phenotypes of monogenic, infectious, and multifactorial disorders may overlap.

### 2.2. Aicardi-Goutières Syndrome and Other Interferonopathies with Brain Involvement

Aicardi-Goutières syndrome (AGS) was first described as an early onset progressive encephalopathy that distinguished itself for high levels (in serum and CSF) of type I IFN, in the absence of prenatal infections [9].

AGS was considered as a “mime” of congenital infections, and this peculiarity has raised attention on the “early-onset encephalopathy—IFN” binomial. Later studies confirmed the neuro-detrimental potential of type I IFN, demonstrating that the chronic production of IFNα from astrocytes leads to the development of the same neuropathological features of AGS [37,38,39,40].

In the last decade, AGS has been classified as the first monogenic type I interferonopathy, displaying pathogenic mutations either in genes associated with defective disposal of nucleic acids (*TREX1*, *RNASEH2A*, *RNASEH2B*, *RNASEH2C*, *SAMHD1*, *ADAR*) or with altered control of the IFN cascade (*IFIH1*) [41]. IFN is closely related to alteration in the gene expression levels of proteins involved in the stability of brain white matter, and downregulation of pro-angiogenic factors and cytokines [40].

After the classification of AGS, other IFN-related disorders have been associated with brain involvement. Even if neurologic manifestations are not part of the typical clinical picture in the STING-associated vasculopathy with onset in infancy (SAVI), some cases have been reported with central nervous system (CNS) involvement [11,42]. Saldanha et al. described a peculiar STING mutation resulting in an unusual SAVI phenotype without cutaneous vasculitis and increased systemic inflammation markers, but with the occurrence of opportunistic infection (potentially life-threatening) and a slow-improving developmental delay [43]. Seo et al. reported the experience of a Korean boy with canonical systemic inflammation and skin lesions, but also brain infarctions revealed by magnetic resonance imaging (MRI) [44].

### 2.3. Therapeutic Experiences

JAK inhibitors are small molecules that inhibit the signaling downstream of several cytokines and are distinguished by an affinity for different JAKs. Nowadays, four molecules received market authorization in humans: tofacitinib (acting on JAK1, JAK2, and JAK3), baricitinib, upadacitinib, and ruxolitinib (JAK1 and JAK2). These drugs have been widely used for clinical trials or as off-label prescriptions, due to their wide spectrum of actions: the effect on JAK1 and JAK2 provides the inhibition of type I and II IFNs and IL-6 signalings, and the blockade of JAK3 (particularly exerted by tofacitinib) significantly reduces the activity of other cytokines and the lymphocyte activation (IL-2, IL-4, IL-7, IL-9, IL-15, and IL-21) [45].

Encouraging results have been obtained in the last years in the treatment of autoinflammatory interferonopathies with JAK inhibitors [46]: e.g., partial improvement in neurologic function in subjects affected by AGS after baricitinib therapy [47], and well-tolerated ruxolitinib treatment for SAVI syndrome, albeit with a significantly increased risk of infections [48,49]. Most authors conclude that further studies are needed to determine the proper balance between the efficacy and safety of JAK inhibitors in these conditions.

Other medications with promising potential for the treatment of interferonopathies have been recently identified in antimalarials, old medications that have been employed for decades in the treatment of rheumatic disorders (as SLE), due to their well-tolerated and strong anti-inflammatory action [50,51]. The antimalarials repositioning is due to the discovery of their mechanism of action, which interferes at many levels with the type I IFN production [52].

### 2.4. A Clinical Experience of Monogenic Interferonopathy: Aicardi-Goutières Syndrome

The first patient was born at 40 weeks of gestation through an uneventful pregnancy and vaginal delivery with good adaptation to extrauterine life from non-consanguineous parents originating in Morocco. Head circumference at birth was 36 cm (60 °C WHO scale). Weight and length were adequate for gestational age. In the 3rd month of life, he was first evaluated for axial hypotonia, persistent archaic reflexes, poor spontaneous movements, mild hypertonia of lower limbs, and secondary microcephaly. At the age of 5 months, his brain MRI showed only a mild white matter involvement, but later on, from the first year of life, a progressive diffuse cortical and subcortical leukoencephalopathy became progressively evident, with basal ganglia, pontine and capsular calcifications and associated diffuse brain atrophy. At this point, AGS was suspected and DNA tested for the *TREX1* gene. The analysis found a missense mutation in exon 7 (A177T) and deletion of exons from 2 to 5 in the *TREX1* gene. The same genetic mutation was found in the older sister, who died at 12 years old of *ab ingestis* pneumonia. Raised IFNα levels in liquor were detected. Autoimmunity screening was negative.

From the age of 4 years, the boy started to present clinical focal seizures with electroencephalogram evidence of left-hemispheric slow waves and disorganized electrical activity. Furthermore, he presented psychomotor delay with severe cognitive impairment, spasticity of upper and lower limbs, and impaired coordination of conjugate gaze. Nowadays, the child is 6 years old, has recently started antispastic therapy with baclofen, and practices physiotherapy 3 times a week. The swallowing function is still preserved allowing a creamy diet. Seizures are well controlled by valproic acid therapy.

### 2.5. Relevance to Multifactorial Disorders: Neuropsychiatric Systemic Lupus Erythematosus

The ever-growing comprehension of the molecular pathogenesis leading to monogenic interferonopathies can reflect on an improved understanding of the pathogenesis of autoimmune/autoinflammatory phenomena in SLE.

Growing evidence supports the view of SLE as a complex autoimmune and inflammatory syndrome, characterized by a wide spectrum of clinical manifestations (skin rash, arthritis, renal and hematological alterations), which may include distinct disease subgroups. Therefore, SLE pathogenesis could depend on the combination of several mechanisms including defective complement function, production of autoantibodies, disorders of lymphocyte tolerance, and nucleic-acid-driven production of IFN [53,54,55,56,57,58].

SLE was considered as the first non-infectious disease associated with an increased type I IFN production. Since the first report in 1979, it has been increasingly proved that type I IFN levels are elevated in the serum and CSF of subjects with SLE [59,60,61]. Moreover, a significant proportion of subjects with SLE may develop symptoms associated with CNS involvement leading to the so-called neuropsychiatric SLE (NPSLE). Although the dysfunctional mechanisms involved in NPSLE are not yet completely unraveled, the peculiar and more frequent damage of NPSLE is represented by cerebral small vessel thrombotic-vasculopathy, which is probably due to the penetration of autoantibodies (mostly antiphospholipid autoantibodies) through the BBB, which triggers pro-inflammatory cytokines (including IFNs) production, prompting and worsening endothelial damage, hence increasing antibodies’ entry [60,61,62,63].

Among the most representative inflammatory cytokines usually detected in NPSLE, the pivotal role of type I IFN in triggering and retaining the cellular damage has been demonstrated by recent studies, which underline that neuropsychiatric symptoms in mouse models of NPSLE could be mitigated by IFNα inhibition [64].

NPSLE insight and management represent a challenge for clinicians so far. The large diversity of neuropsychiatric symptoms, and their nonspecific manifestations (e.g., from mild headaches and cognitive deficits to severe seizures and stroke), make difficult both the direct attribution to SLE, and the discrimination between primary or secondary SLE events (e.g., high-dose corticosteroid treatment). Moreover, the incomplete knowledge of the pathogenetic mechanism, along with the lack of a “gold-standard” diagnostic method (NPSLE could be not confirmed by specific laboratory or imaging findings), makes the therapeutic choice even more difficult [65,66].

Given the great heterogeneity of neurological symptoms (in terms of type and severity), it could be of great interest to investigate the possible role of type I IFN for the stratification of subgroups of patients with NPSLE, to evaluate the therapeutic potential of targeting IFN inflammation.

## 3. From Monogenic to Multifactorial: TNFα-Related Brain Disorders

### 3.1. DADA2 and Monogenic Vasculitis Syndromes with Cerebral Involvement

Vasculitides are intended as a heterogeneous group of disorders due to an inflammation of the blood vessels that can result in vascular injury of involved tissues/organs. The wide spectrum of clinical symptoms is related to the causes of the inflammation process, and type and location of the involved vessels (e.g., vasculitis occurring close to the skin is prevalently marked by different types of rash with urticaria, chilblain-like lesions, whereas vasculitis of mucous membranes causes aphthous ulcers).

The term vasculitis usually refers to systemic vessels’ inflammation (differing from isolated events, non-systemic vasculitis), that can develop as a spontaneous disease, as a reaction to drugs or chronic infections, or as part of complex autoimmune diseases such as rheumatoid arthritis (RA) or SLE [67,68,69].

The homeostatic function of endothelial cells in vascular tone/structure regulation is exerted by holding an “anti-inflammatory environment”, by the downregulation of adhesion molecule, preventing the migration and adhesion of leukocytes, and platelet aggregation [70,71]. However, the presence of pathogenic antibodies triggers the “endothelial activation” via the pathophysiological crosstalk between brain macrophages, platelets, and endothelial cells, leading to a vicious circle of immune cells activation and proinflammatory mediators secretion, mostly type I IFN for SLE and RA, and tumor necrosis factor-α (TNFα) [2,3,72,73,74,75,76,77].

Vasculitis can affect every district of the body. The involvement of vessels of the central and/or peripheral nervous system may cause thrombosis, vasospasm, aneurysms, and hemorrhages, with a variable clinical expression of stroke episodes [78,79].

In 2014, it was described the first monogenic early-onset stroke and vasculopathy disease, characterized by neurovascular manifestations (ischemic and/or hemorrhagic stroke) and systemic inflammation with mild immunodeficiency, that can be confused with other systemic rheumatologic disorders. The deficiency of adenosine deaminase 2 (DADA2) is related to a mutation in the *CECR1* gene (cat eye syndrome chromosome region, candidate 1) encoding for the ADA2 protein, which is mainly present in myeloid cells as a secreted protein. Together with the ubiquitously expressed ADA1 protein, ADA2 regulates purine metabolism in a non-redundant way. The higher affinity of ADA1 for substrates explains the more severe combined immunodeficiency (SCID) phenotype in subjects with *ADA1* mutation [80,81].

**Figure 2:**
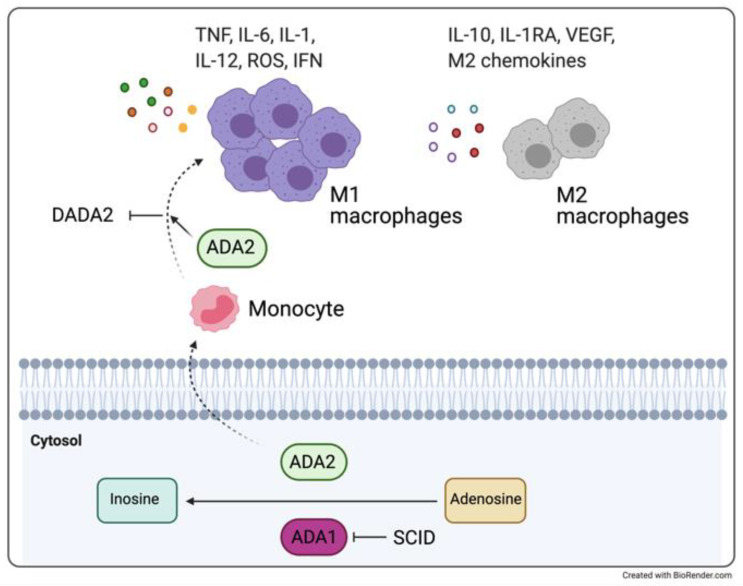
Like ADA1, ADA2 is involved in the conversion of adenosine into inosine. On one hand, a deficit of ADA1 protein is responsible for the development of severe combined immunodeficiency (SCID), on the other hand, ADA2 deficiency is associated with reduction of M2 macrophages and polarization towards M1 macrophages, with consequent increased production of proinflammatory cytokines and endothelium damage and fibrosis, and DADA2 disease. Figure adapted from [87].

Even though endothelial cells being destroyed in co-culture with monocytes of patients with DADA2 is described [12], the pathologic mechanism linking ADA2 deficiency, and the endothelial damage is not yet completely unraveled. Conversely, it is known that ADA2 has a crucial role in balancing the repertoire of pro-inflammatory (M1) and anti-inflammatory (M2) monocytes [12,82,83]. Indeed, ADA2 deficiency is associated with the reduction of M2 macrophages and polarization towards M1 macrophages, with consequent increased production of proinflammatory cytokines (enhancing TNFα and IFNs release), endothelium damage, and fibrosis [84,85,86] (Figure 2).

### 3.2. Therapeutic Experiences

Despite partially successful results being obtained with immunosuppressant drugs (e.g., azathioprine, cyclosporine, tacrolimus, cyclophosphamide, methotrexate) [12,13,80,82], the anti-TNFα agents (etanercept, adalimumab, and infliximab) are currently considered the drugs of choice for DADA2 treatment: the positive long-term effect in controlling the inflammatory and vascular manifestations have been observed in independent studies [13,80,88].

Recent observations from different cohorts of patients with DADA2 suggested a possible involvement of type I IFN in the disease, however its role as a therapeutic target remains unexplored [89,90].

Considering that ADA2 protein is secreted from bone marrow-derived myeloid cells, hematopoietic stem cell transplantation could be reasonably considered as a definitive treatment, allowing the correction of both the inflammatory and immunodeficiency-related phenotypes [91]. However, this approach is currently proposed to subjects whose disease is associated with significant marrow failure and immunodeficiency [91,92,93].

### 3.3. A Clinical Experience of a Monogenic Disorder Associated with TNF-Related Inflammation: DADA2

The patient is a girl who was admitted to our institute at the age of five because of circular, raised, painful reddish skin lesions on the legs, which were attributed to indeterminate vasculitis. In her first years of life, she had presented recurrent episodes of unexplained fever with increased acute phase reactants. After histological examination of skin lesions, she was also diagnosed with celiac disease and put on a gluten-free diet. Intravenous Immunoglobulins and methotrexate failed to control the disease that only responded to glucocorticoid therapy, although with frequent relapses on therapeutic tapering.

In the following two years, the mother reported several episodes of fever, vasculitis. At 7 years, the girl presented diplopia with eyelid ptosis and elevation of acute phase reactants. She also presented a picture of livedo reticularis on her legs. The girl appeared in good general condition, oriented, alert, without any walking or speech disturbance. Normal heart activity. Neurological examination evidenced the involvement of the oculomotor nerve with disturbance of the external oblique muscle. MRI revealed a small ischemic lesion of the mesencephalic cap in the right paramedian region that did not take the contrast medium and was likely due to a previous stroke. Familial history revealed a high recurrence of vascular accidents and autoimmunity on the maternal side. The mother presented multiple miscarriages; an aunt died at the age of 48 of cerebral hemorrhage, after a story of fatigue, Raynaud’s phenomenon, miscarriage, sudden loss of sight and hearing. Another maternal aunt was struck by sudden blindness and unilateral deafness; a cousin had a diagnosis of polyarteritis nodosa. Furthermore, the 10-year old patient’s brother had presented with an intracranial hemorrhage of non-traumatic origin.

Laboratory examination for antiphospholipid antibodies and other vasculitis-related autoantibodies were negative.

The girl was successfully treated with methylprednisolone boluses and cyclophosphamide and discharged in antiaggregant therapy.

Only some years later, when the genetic test for DADA2 became available, she and her brother would be found affected with DADA2 deficiency based on the detection of the homozygous T360A mutation in *CECR1*. Of note, some expression of the disease was variably recorded also in the heterozygous relatives. Given the emerging knowledge on DADA2, an anti-TNFα therapy was proposed afterward.

### 3.4. Relevance to Multifactorial Models: Behçet’s Disease-Associated Vasculitis

Another example of advantage in describing complex diseases based on the level of similarity with prototypical monogenic disorders is represented by BD, considered as a chronic multi-organ disorder which is hardly ranked as autoimmune or autoinflammatory systemic vasculitis. BD usually occurs with mucocutaneous lesions (urogenital ulcers) and variable patterns of vascular, skin, eyes, articular and gastrointestinal manifestations. The specific etiology is unknown, even if a hypothesized role for infectious agents (virus or bacterium) in the inflammation initiation and/or exacerbations at the vascular and gastrointestinal surfaces has been described [94].

The possible contribution of several pathogenic mechanisms may account for the shaping of distinct forms of the disease [95,96], bringing some patients to develop a rheumatologic condition like SLE or Sjögren syndrome, while others could remain without a definite diagnosis. Indeed, considerable overlaps could be described between some cases of pediatric BD and SLE, due to causative genes that may underlie both conditions, as in the case of A20 haploinsufficiency (*TNFAIP3* gene). A20 haploinsufficiency may, however, show distinctive clinical features, such as early-onset in children, familial occurrence, recurrent fever attacks, gastrointestinal involvement, and infrequent ocular involvement, which can influence follow-up and therapeutic choices [97,98].

The BD’s chronic vasculitis involves vessels of any size (both arteries and veins) whose endothelia are damaged by the over-production of reactive oxygen species by the infiltrating neutrophils, resulting in impaired coagulation and thrombosis [99,100]. Several studies support a crucial role for an abnormal lymphocyte and monocyte activation with chronic release of TNFα in BD vasculitis [101,102,103].

Neurological involvement (neuro-BD) is relatively uncommon but occurs with serious manifestations going beyond headaches, and including meningitis, hemiplegia, cerebral venous sinus thrombosis, intracranial hypertension, and psychiatric symptoms (including personality changes) [104,105]. Neuro-BD is still a challenging diagnosis because other conditions may lead to a similar clinical picture (e.g., viral infections, strokes) [106]. Even if a large variety of medications has been proposed for BD therapy (colchicine, glucocorticoids, immunosuppressants, biologics), TNFα inhibitors have been proven particularly useful to treat cases with ocular and CNS involvement [107,108,109].

## 4. From Monogenic to Multifactorial: IL-1β-Related Brain Disorders

### 4.1. Cryopyrin-Associated Periodic Syndrome and Aseptic Meningitis

The inflammatory cryopyrin-associated periodic syndrome (CAPS) encloses three phenotypes with a diverse grade of severity: the mildest familial cold autoinflammatory syndrome (FCAS), Muckle-Wells syndrome (MWS), and the most severe neonatal-onset multisystem inflammatory disease (NOMID, also known as CINCA, chronic infantile neurological cutaneous and articular syndrome). Clinical features of CAPS syndromes consist of both systemic inflammation and fevers and local manifestations at the skin, joints, muscles, eyes, and CNS, albeit each sub-phenotype could occur with peculiar clinical features [15,110,111].

**Figure 3:**
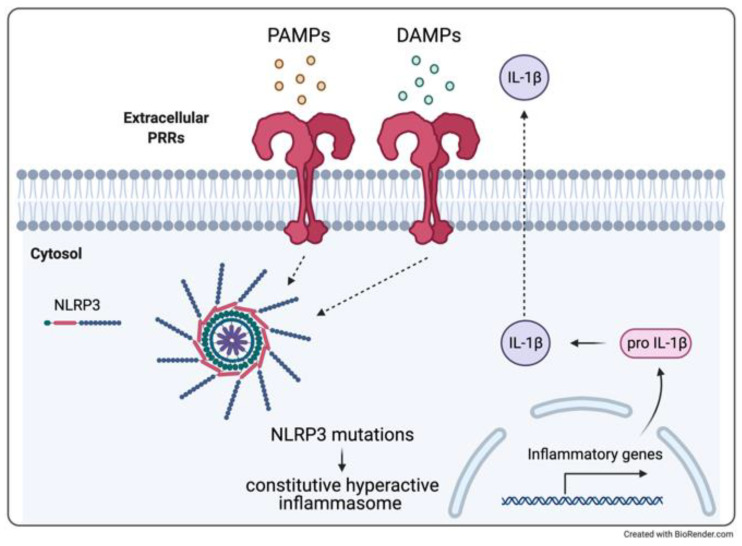
Inflammasome activation. Defects in the *NLRP3* gene result in constitutive activation of the inflammasome and a consequence dysregulation of IL-1β production.

CAPS are related to a gain-of-function mutation of the *NLRP3* gene coding for cryopyrin, which is fundamental for the intracellular complexes known as inflammasomes, a key component of the innate immune system (Figure 3). Defects in *NLRP3* result in a constitutive hyperactive inflammasome with a dysregulated release of IL-1β, one of the major inflammatory mediators which is responsible for the systemic inflammatory “trio” of cutaneous, rheumatologic, and neurological symptoms [112,113,114,115].

CNS involvement ranges from milder headaches to progressive hearing loss, chronic aseptic meningitis, and mental retardation. Considering that the strong inflammatory action of IL-1β is suggested to be related to the typical neurodegeneration of MS [116], it is highly possible that this cytokine is strictly related also to the brain lesions in patients with CAPS syndrome [117].

### 4.2. Therapeutic Experiences

While specific drugs are under development to directly inhibit NLRP3, treatments for CAPS (as for other autoinflammatory syndromes) are currently focused on IL-1 inhibitors: anakinra (the first anti-IL-1 drug clinically available), a recombinant form of IL-1RA which binds IL-1 receptor preventing both IL-1α and IL-1β binding and signaling, and canakinumab, a monoclonal antibody that selectively blocks IL-1β. Whilst anakinra is a BBB permeant drug, canakinumab is thought to reach the CNS only in inflammatory conditions.

The efficacy and safety of anakinra and canakinumab for CAPS treatment has been widely reported, along with little side effects (mostly infections) and rapid improvement of clinical features [114,118,119].

### 4.3. Clinical Experience of Monogenic Model: CINCA

A 7-year-old girl was referred to our attention because of a complex clinical history, with an urticarial skin rash starting soon after birth, subsequently accompanied with daily fever spikes. Her symptoms could be attenuated by treatment with glucocorticoids but relapsed on drug discontinuation. From the age of 2 years, she also developed knee arthritis. Laboratory data always showed increased acute phase reactants. During follow-up, she also presented headaches, which were associated with papilledema at eye examination and mild cerebral atrophy at brain MRI. Furthermore, hearing tests showed perceptive deafness. Her facies was peculiar for frontal bossing and low bridge nose. Furthermore, she presented an overgrowth of the patella, which is a highly supportive sign for CINCA (chronic cutaneous, neurological, articular syndrome). Only some years later, a molecular diagnosis would be possible (heterozygous causative *NLRP3* mutation in somatic mosaicism) and a specific treatment could be initiated with anakinra, a biologic IL-1 blocking medication that allowed, for the first time, complete control of autoinflammatory symptoms, together with the disappearance of the rash. Unfortunately, the treatment could not cure deafness.

Subsequently, in other cases from our series and from the literature, an early start of IL-1 blocking therapy was shown able to prevent or even revert most of the disease-related complications, including deafness [120].

### 4.4. Relevance to Multifactorial Neurologic Inflammatory Disorders

Febrile infection-related epilepsy syndrome (FIRES) appears as an acute encephalopathy with unknown etiology and develops in previously healthy children and adolescents (3–15 years old) after a simple febrile illness. FIRES is characterized by recurrent focal seizures that lead to a decline in memory, cognition, and behavior. Some patients develop psychiatric disorders and/or motor disability, while others can evolve to a vegetative state, or even death [121,122]. Antiepileptic drugs are of little efficacy. The pathogenic mechanism underlying FIRES is not unraveled, and no infectious agent is generally identified, but the finding of proinflammatory cytokines in CSF was supportive of immune-mediated pathogenesis [123,124].

Hence, the potential of immunomodulatory therapies is increasingly considered, and certain strategies to modulate the immune system have been applied with anakinra or tocilizumab [125,126,127].

## 5. Conclusions

Neurological disorders have been evermore associated with an immunopathological cause. The early detection of inflammatory-driven brain manifestations is fundamental for the diagnostic process (mainly in pediatrics), encouraging the choice of the most appropriate therapeutic strategy to mitigate potentially detrimental complications. Knowing how the immune system can harm the brain helps to elaborate a strategy to counteract the involved dysfunctional mechanism. It is of paramount importance to correctly identify the precise deranged pathway in order to use the right molecule, e.g., a monoclonal antibody or a receptor pharmacological inhibitor, capable to block an exaggerated response or enhance a defective regulating function. CSF and blood cytokine profiling may represent a valuable aid supporting diagnosis and treatment.

Monogenic immune-dysregulations with neurological manifestations could be considered as “models” to unravel and deepen the underpinning pathogenic mechanisms that may be shared by multifactorial neuro-disorders, which can, therefore, benefit from targeted treatment with specific immuno-modulators.

## Figures and Tables

**Table 1:** Medications for immune-mediated brain diseases.

Target	Medications	Monogenic Disorders (Key Neurological Symptoms)	Multifactorial Disorders
Type I IFN	JAK inhibitors (baricitinib, ruxolitinib, tofacitinib), antimalarials	Type I interferonopathies (seizures, leukodystrophy, headaches)	SLE
TNFα	Etanercept, adalimumab, infliximab	DADA2 vasculitis, strokes	Neuro-BD
IL-1 receptor	Anakinra	CAPS, aseptic meningitis, *pseudotumor cerebri*	FIRES

Legend: IFN, interferon; SLE, systemic lupus erythematosus; TNFα, tumor necrosis factor-α; DADA2, deficiency of adenosine deaminase 2; BD, Behçet’s disease; IL-1, interleukin-1; CAPS, cryopyrin-associated autoinflammatory syndrome; FIRES, febrile infection-related epilepsy syndrome.

**Table 2:** Monogenic diseases with immune-mediated involvement of the central nervous system.

Disease	Gene(s)	Dysregulated Signaling Pathway	Principal Clinical Features	Neurological Involvement	Inheritance
AGS [9,10]	*TREX1* *RNASEH2* *(A, B, C)* *SAMHD1* *ADAR* *IFIH1*	Type I IFN	Intermittent fevers, hepatosplenomegaly, chilblains	Progressive cerebral atrophy, leukodystrophy, intracranial calcifications, chronic CSF lymphocytosis, progressive psychomotor retardation, seizures	AD/AR
SAVI [11]	*STING/TMEM173*		Severe skin lesions (face, ears, nose, digits), interstitial lung disease, livedo reticularis, Raynaud phenomenon, recurrent fevers	Developmental delay, brain infarctions	AD
DADA2 [12,13]	*CECR1/ADA2*	TNFα	Recurrent fevers, systemic vascular inflammation (skin ulcerations, strokes), Raynaud phenomenon	Neurologic sequelae of stroke, headaches, ataxia	AR
CAPS [14,15]	*CIAS1/NLRP3*	IL-1β	Fever, rash, arthralgia	Chronic meningitis with headaches, deafness and blindness (partial or complete), mental retardation	AD

Legend: AGS, Aicardi-Goutières syndrome; IFN, interferon; CSF, cerebrospinal fluid; AD, autosomic dominant; AR, autosomic recessive; SAVI, STING-associated vasculopathy with onset in infancy; DADA2, deficiency of adenosine deaminase 2; TNFα, tumor necrosis factor-α; CAPS, cryopyrin-associated autoinflammatory syndrome; IL-1β, interleukin-1β.

## Data Availability

Data sharing not applicable.
